# Genomic Insights and Bioconversion Potential in the Black Soldier Fly (*Hermetia illucens*): Current Advances and Future Directions

**DOI:** 10.3390/insects17010070

**Published:** 2026-01-07

**Authors:** Joana Oliveira, Leonardo Gaston Guilgur, Ricardo Assunção, Daniel Murta, Alexandre Trindade

**Affiliations:** 1 Egas Moniz Center for Interdisciplinary Research (CiiEM), Egas Moniz School of Health & Science, 2829-511 Almada, Portugalatrindade@egasmoniz.edu.pt (A.T.); 2 Organismal Metabolic Physiology, Gulbenkian Institute for Molecular Medicine (GIMM), 1649-028 Lisbon, Portugal; 3 Food and Nutrition Department, National Institute of Health Dr. Ricardo Jorge, 1649-016 Lisbon, Portugal; 4 Ingredient Odyssey SA—EntoGreen, 2005-002 Santarém, Portugal; 5 Thunder Foods SA, 2005-332 Santarém, Portugal

**Keywords:** black soldier fly, bioconversion, black soldier fly genetics, genome editing

## Abstract

The global population continues to rise, placing increasing pressure on the agri-food sector and leading to the accelerated generation of urban organic waste, factors that collectively intensify climate stress and environmental instability. Insects are recognised for their remarkable capacity to transform substrates into valuable products, with the black soldier fly larvae (BSFL) emerging as one of the most efficient and widely utilised species for this purpose. Beyond recycling organic matter, BSFL can also mitigate microbial contamination, effectively reducing bacterial and fungal loads in waste substrates. Understanding and manipulating the genome could provide tools to improve BSFL bioconversion process and contribute to sustainability. In this review, we provide an overview of recent advances in black soldier fly genomics and genome-editing technologies. Although research in this subject remains limited, recent studies have clarified its origin, characterised its genome, and established the foundation for targeted genetic improvements to enhance by-product conversion, nutrient recovery, and environmental sustainability.

## Simple Summary

The black soldier fly (*Hermetia illucens,* BSF) is an efficient converter of organic by-products into high-value outputs such as animal feed and organic fertilisers, thereby mitigating environmental pressures by reducing pollution and greenhouse gas emissions. This review summarises the current knowledge on the species’ genetic origin, genome composition, and adaptations that underpin its exceptional bioconversion capacity. Recent genomic and transcriptomic studies have identified key genes involved in digestion, immunity, and environmental adaptation, providing a genetic framework for enhancing conversion efficiency. Furthermore, genome-engineering technologies now enable precise genetic manipulation and the development of transgenic lines with improved traits. These breakthroughs open new opportunities to optimise waste upcycling, nutrient recovery, and sustainable biomass production within a circular economy framework.

## 1. Introduction

The United Nations expects that the global population will reach 9.7 billion people by 2050, emphasising the growing demand for sustainable food systems [1]. With this rate of growth, the agri-food sector will need to increase its output to meet global demand. It is well known that agriculture and livestock farming are major contributors to environmental pollution. In the European Union (EU), agriculture accounts for about 93% of ammonia emissions; alongside livestock farming, which not only contributes to this emission but also produces methane [2]. Simultaneously, urban organic waste (UOW), particularly food waste, is a growing concern, with 2.01 billion tonnes produced annually, expected to rise to 3.4 billion tonnes by 2050 [3].

Traditional UOW management methods, including landfilling, incineration, composting, and anaerobic digestion, have limitations, such as the emission of greenhouse gas emissions and undesirable odours [3,4,5,6,7]. All these factors pose risks to human and animal health, lead to famine, and increase poverty, affecting the lives of the entire human population. Thus, it is crucial to find innovative solutions for UOW management.

Insects are recognised for their high efficiency in bioconverting organic matter. They also represent a potential alternative source of protein for food and feed [8,9].

The black soldier fly (BSF; *Hermetia illucens*) belongs to the order Diptera, family *Stratiomyidae*, subfamily *Hermetiinae* [10]. It is native to tropical, subtropical, and temperate regions of America [11]. Its life cycle includes five stages: eggs, larvae, prepupae, pupae, and adults. The BSF has a short life cycle lasting between 40 and 43 days [12,13]. The larval stage lasts around two weeks, at optimal conditions [14] and is the only stage where the BSF feeds, with larvae voraciously consuming organic matter [15]. The larvae of the black soldier fly (BSFL) are the most used insect for bioconversion and can significantly reduce large volumes of organic substrates [16]. Despite these organic substrates being contaminated with a variety of biological and chemical contaminants, some studies indicate that BSFL can eliminate bacteria such as *Salmonella* spp. and *Escherichia coli* [17,18,19]. Furthermore, BSFL are capable of reducing over 90% of CO_2_ and 94% of NH_3_ emissions [20,21,22,23,24]. The bioconversion of organic substrates by BSF aligns with the circular economy concept, where organic by-products are reintegrated into the agri-food chain, preserving their value for as long as possible [25]. Consequently, insect farming offers both economic benefits and environmental advantages.

Despite widespread industrial use of BSF, its genetic and molecular basis remains poorly understood, representing a critical knowledge gap for improving bioconversion efficiency and industrial applications. Traits related to larval performance in bioconversion are influenced by the species’ genetic background [26]. Understanding the genetics of these traits remains limited, as BSF is still genetically underexplored compared to well-known insect models such as the fruit fly (*Drosophila melanogaster*) [27].

The bioconversion capacity of BSF offers an environmentally friendly strategy to transform approved organic by-products into valuable biomass, which can be used for, e.g., feed production, organic fertilisers, biofuel, pharmaceuticals and even cosmetics [28,29,30,31]. Optimising this process through genetic and molecular insights could further enhance conversion efficiency, nutritional quality, and biosafety.

**Figure 1:**
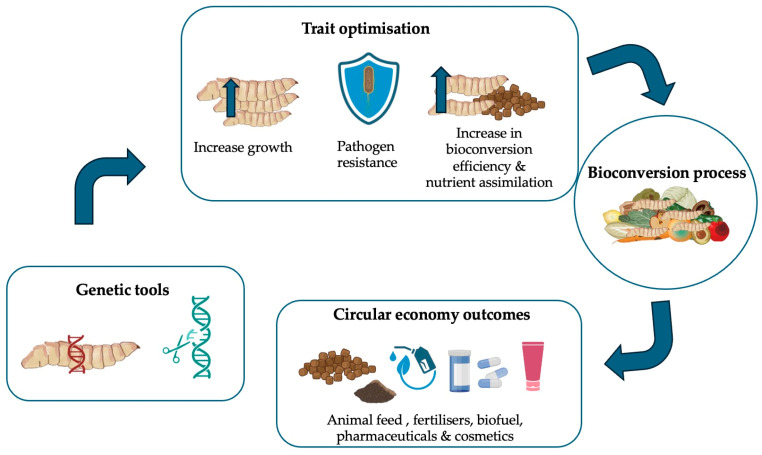
Conceptual framework linking black soldier fly genomics to bioconversion performance and circular economy outcomes. Authors’ original.

Figure 1 provides a conceptual overview linking black soldier fly genomics to bioconversion performance and circular economy outcomes, highlighting how genetic knowledge underpins industrial optimisation and biosafety.

The aim of this review is to provide an overview of the current state of knowledge concerning the genetic profile of BSF, with a particular emphasis on the recent advancements in genome analysis and editing and how it can drive the future development of insect biotechnology.

## 2. The Black Soldier Fly Origin

Understanding the origin of the black soldier fly is key to genetics and industrial applications. The origin of the BSF has long been debated, but phylogeographic studies provide strong evidence for a South American origin. A study by Kaya et al. [32] genotyped and analysed 2863 BSF individuals from 150 populations across 57 countries worldwide. Their data revealed genetic hotspots in South America, indicating that BSF likely originates from this continent. This was further supported by Guilliet et al. [33], who analysed 677 *COI* sequences from BSF across five continents, identifying 52 haplotypes—including 10 major haplotypes—by sequencing 59 mitochondrial genomes. In their study, the authors found 30 haplotypes among 55 sequences in South America, thereby confirming the South American origin of the BSF. In addition to establishing the origin, Guilliet et al. also identified evidence of three introductions of BSF in Australia and three in Asia. Seven haplotypes were detected in Europe, suggesting at least seven introductions of the species to this continent. Four haplotypes were also identified in Africa. Mapping native diversity and tracing introductions highlights lineages that may carry alleles for faster growth, broader substrate tolerance, disease resistance, or altered nutrient profiles, offering valuable material for selective breeding and crossbreeding [34,35]. Concerning the commercial strain, the BSF populations used for breeding in Europe and North America all originate from a common strain [32]. Guilliet et al. found no South American relatives in the commercial strain. It has been proposed that the commercial strain originated in North America [33], but determining its exact origin could be challenging. In this context, origin-aware genomics is crucial for biosafety, preventing commercial lines from compromising native populations or ecosystem integrity [36].

## 3. The Black Soldier Fly Genetic Profile

Despite growing research interest, the BSF genome has not been extensively studied. Recently, a study by Zhan et al. [37] sequenced the entire BSF genome. They reported that the BSF genome is larger than that of other dipteran species (1102 Mb), containing 16,770 genes. About two-thirds of the genome consists of transposable elements and other repetitive non-coding DNA. The mean intron size in BSF is reported to be the second longest among dipteran species. The authors also found that half of the BSF genes are common to all dipteran species they analysed. They further showed that the BSF genome encodes 797 Brachycera-specific genes and 1798 species-specific duplicated genes. Most of these genes are expressed during the late larval stage. Since larvae feed on organic matter, this gene duplication could be involved in shaping this particular BSF trait.

Generalovic et al. [38] produced a high-quality chromosome-scale genome assembly, annotating over 16,000 protein-coding genes in six autosomes and the X chromosome. More recently, high-throughput sequencing has been used to characterize the complete mitochondrial genome of BSF (*Hermetia illucens*) from different isolates. Albalawneh et al. [39] sequenced the Jordan_1 BSF isolate, revealing a mitogenome of 15,699 bp with a GC content of 28.2%, comprising 13 protein-coding genes, 22 tRNA genes, and two rRNA genes. Comparative analysis with reference mitogenomes identified one insertion/deletion (*InDel*) and 57 single-nucleotide polymorphisms (*SNPs*). Similarly, Qi et al. [40] reported a mitogenome length of 15,698 bp, containing the same gene content and a non-coding control region. This study additionally reconstructed the phylogenetic relationships of *H. illucens* with 12 related species, confirming its taxonomic status and providing valuable information for mitochondrial genomics and evolutionary studies. Moreover, Ståhls et al. [41] examined BSF mitochondrial genetic diversity across 39 countries using *COI* sequences. They found surprisingly high intraspecific variation (up to 4.9%) in mitochondrial barcoding genes, with 56 distinct *COI* haplotypes identified globally. Despite this mitochondrial diversity, nuclear markers (*ITS2* and *28S* rDNA) were invariant, and laboratory hybridization experiments confirmed reproductive compatibility. This study highlights the complex mitochondrial phylogeography of BSF and underscores the importance of considering mitochondrial genomic variation in population, phylogenetic, and breeding studies.

### 3.1. BSF Affinity for Organic Substrates

The BSF is one of the most interesting insects for recycling and reuse of organic by-products, converting them into animal feed and/or organic fertiliser [42].

Zhan et al. [37] discovered that BSF uses a common set of genes to respond to different types of organic substrates, with 326 genes being expressed in larvae fed various organic substrates. These genes were enriched in 13 biological pathways, with ribosome genes being the most prominent. Since the ribosome is responsible for protein synthesis, the authors suggest that BSF’s effectiveness in bioconversion of organic substrates could be linked to its ribosomal function. Genes related to digestive functions were also highly enriched. Given the central role of ribosomes in protein synthesis, this enrichment likely supports the rapid larval growth and high biomass yield characteristic of BSF-based bioconversion systems. However, direct quantitative correlations between ribosomal gene expression levels and substrate reduction rates or feed conversion efficiency have not yet been established.

Transcriptomic studies have further revealed that BSFL respond differently to substrate types at the molecular level. For example, larvae fed swill versus pig manure exhibited distinct expression patterns in genes associated with energy metabolism, amino acid metabolism, redox reactions, and stress responses [43]. These results indicate a high degree of transcriptomic plasticity, suggesting that substrate composition can modulate BSFL growth, metabolism, and stress physiology. Moreover, Zhu et al. [44] identified key genes involved in pyruvate and acetyl-CoA metabolism, fatty acid biosynthesis, and triacylglycerol formation, which likely contribute to substrate-driven fat accumulation in larvae.

Silvaraju et al. [45] analysed the gut bacterial communities of two genetically distinct BSF lines, a wild-type (WT) and a lab-adapted (LD) line and found distinct microbial profiles between them. Their findings indicate that the gut microbiota of BSF larvae is influenced by genetic factors, highlighting the interplay between host genetics and microbial composition in shaping larval physiology and substrate conversion efficiency. Other studies further emphasise that gut microbiota composition is highly substrate-dependent and can enhance nutrient assimilation, pathogen resistance, and bioconversion efficiency [43,46,47,48,49]. These insights highlight the host-microbiome interactions in determining BSFL performance across different substrates.

### 3.2. BSF Environmental Adaptation

Considering the affinity of BSFL for organic matter, it is likely that this insect interacts with various pathogens. Therefore, the immune system of BSF has probably adapted to dealing with pathogenic microbes.

The antimicrobial activity of the BSFL has already been studied [18,19,50,51,52,53]. Besides antimicrobial peptides (*AMPs*), there are peptidoglycan recognition proteins (*PGRPs*), Gram-negative binding proteins (*GNBPs*), and *phenoloxidase* coding genes within the BSF genome that contribute to this antimicrobial activity.

The BSF genome encodes 31 secreted *PGRPs* far more than in other dipteran species. These proteins play essential roles in regulating signaling pathways during bacterial infection [37].

The expansion of the repertoire of recognition molecules, as in the case of *GNBPs*, which are hemolymphatic proteins involved in activating the *Toll* pathway, may represent a strategy for responding to a wide range of pathogens. Consistent with this, 16 *GNBP*-encoding genes were identified in the BSF genome, once again a number markedly higher than that observed in other dipteran species [37].

*AMPs* are primarily synthesised in the fat bodies and blood cells of insects [53]. There are 57 *AMPs* encoding genes in the BSF, with the *cecropin* family constituting the majority [37,54]. The mechanism of action of cecropins is to induce lysis in the cell membranes of both Gram-negative and Gram-positive bacteria [55]. 

Vogel et al. [54] identified 26 genes that encode *defensin AMPs*. Defensins cause membrane disruption, mainly in Gram-positive bacteria, by affecting the membrane electrostatic charge [55].

*Sarcotoxin* and *stomoxyn* genes were also expressed in BSFL [56]. The identification and cloning of *stomoxynZH1*, followed by its expression in *Escherichia coli* (*Trx-stomoxynZH1*), demonstrated antimicrobial activity against both Gram-negative and Gram-positive bacteria, as well as filamentous fungi [56].

*Phenoloxidase* genes were also identified by Vogel et al. [54]. The phenoloxidase system defends against Gram-positive and Gram-negative bacteria, as well as fungi and viruses [55].

Additionally, recent evidence shows that the BSFL immune system is complemented by its gut microbiome. Host-mediated recruitment of beneficial environmental microbes enhances larval survival, pathogen suppression, and substrate bioconversion [48,49]. Functional microbial consortia, including *Enterococcus*, *Bacillus*, and *Lactobacillus*, contribute to disease resistance and metabolic support, demonstrating the integration of host genetics, immune response, and microbiome function as a strategy for environmental adaptation and waste valorisation.

## 4. Genome Editing to Improve Performance Traits in the BSF

As highlighted above, the BSF plays a central role in valorising organic by-products into resources for feed and fertilisers. However, there are still some challenges regarding BSFL’s usefulness for production purposes. These challenges range from limited options of organic substrates approved as insect feed to issues with the traceability of the bioconversion process and a knowledge gap concerning BSFL’s biological information. Genetic manipulation offers promising solutions to address these limitations. By enabling targeted modification or regulation of key genes, biotechnology can be used to enhance desirable traits. Studies on this subject have been widely conducted in some model insects, especially in *Drosophila melanogaster* [57,58,59].

CRISPR/Cas9 genomic techniques have become particularly significant. This method involves two components: a guide RNA to target a specific gene and Cas9, an endonuclease that causes a double-stranded DNA break, enabling modifications to the genome [60]. Studies of this technology in insects are limited, having been applied to groups including Diptera, Lepidoptera, Coleoptera, Orthoptera, and non-insect arthropods [61]. In insects, the first use of this protocol was in *Drosophila melanogaster*, followed by *Bombyx mori*; afterwards, it was employed in various flies, mosquitoes, moths, and butterflies [59,61].

In a recent study, the authors developed an approach to improve the efficiency of organic matter consumption by BSFL [37]. These authors aimed to extend the larval stage, which is the only stage where the fly feeds, by mutating the prothoracicotropic hormone (PTTH) gene, which influences molting and metamorphosis by mimicking the signaling cascade that leads to the biosynthesis and release of ecdysone [37]. Zhan et al. [37] found that both body size and weight were larger in the mutant larvae. Because the larval stage is the only feeding stage, extending its duration directly increases biomass yield per unit of substrate, a key indicator of bioconversion performance. Nonetheless, data quantifying substrate reduction efficiency or nutrient recovery following *PTTH* disruption are still limited. Recently, a piggyBac transposon system was used to establish both transient and germline-stable, effective transgenic BSF [62]. This setup employed the endogenous *Hiactin5C* promoter to drive expression of a hyperactive variant of the *piggyBac* transposase, which showed higher transformation efficiency in BSF than the non-optimised transposase. Using this transgenic system, the authors successfully generated stable BSF lines expressing Green Fluorescent Protein (GFP) under the control of the robust and ubiquitous *IE1* promoter. It is worth noting that this *IE1* promoter has previously been utilized to drive transgene expression in model insects such as *Drosophila melanogaster* and *Bombyx mori* [63]. Green fluorescence was observed in larvae and in the mouthparts and regions of the abdomen and legs of adults. The germline transformation rate ranged from 8% to 18%, which was higher than the 0% to 10% transformation rate reported for most insect species. Additionally, these authors demonstrated that the endogenous *HiU6:1* and *HiU6:2* promoters are the best candidates to drive expression of shRNAs that decrease the expression of a reporter gene, supporting further CRISPR/Cas9 development.

Moreover, using microinjection of *piggyBac* mRNA and donor plasmids, stable transgenic BSF lines can now be generated and screened in under two weeks, with transformation rates of 30–33%, and early confirmation of transgene transmission is possible just three days after outcrossing [64]. The establishment of fluorescent BSF lines enables direct monitoring of larval biomass flow and substrate-to-product traceability, addressing a major biosafety and process control limitation in large-scale bioconversion facilities. A recent study by Kou et al. established a binary transgenic CRISPR/Cas9 system enabling efficient BSF genome editing. The authors identified and functionally characterized several germline-specific promoters (*nanos*, *vasa*, and *exuperantia*), demonstrating that *Hiexu* and *Hinos* exhibit the strongest activity in both ovaries and testes. Using a *piggyBac*-based germline transformation system, they generated transgenic BSF lines expressing Cas9 under these promoters, which displayed normal development and fertility. When the Cas9-expressing lines (*Hiexu-Cas9* and *Hinos-Cas9*) were crossed with individuals carrying sgRNAs targeting the *white* marker gene, the researchers achieved highly efficient germline mutagenesis. Moreover, targeting the *vestigial* gene produced completely wingless and sterile adults, incapable of flight or mating, offering a safe and controllable strategy for industrial mass rearing. Although this modification does not directly enhance bioconversion efficiency, it provides an important biosafety tool that facilitates the industrial deployment of genetically improved BSF strains without increasing ecological risk. This work represents a breakthrough in BSF functional genomics, providing robust tools for precise genome editing and targeted genetic improvement [65].

Furthermore, Gunther et al. [66] recently engineered transgenic BSF capable of producing carotenoids, specifically provitamin A compounds, by inserting two key carotenoid biosynthetic genes, *CarRA* and *CarB*, into the fly genome. This innovation addresses a major nutritional limitation of BSFL, which are naturally high in protein but lack fat-soluble vitamins such as vitamin A. By enabling the flies to synthesise carotenoids internally, rather than relying on dietary supplementation, this approach supports closed-loop production systems and reduces the need for specialized feed inputs. By increasing the micronutrient value of the larval biomass without altering the substrate, this strategy improves nutrient recovery efficiency from organic waste streams, expanding the functional output of BSF bioconversion systems beyond protein and lipid production.

In parallel, genome editing could be directed towards enhancing digestive efficiency, expanding the range of substrates that larvae are able to bioconvert. Notably, recent work employing CRISPR/Cas9-mediated genome editing generated a BSF strain with enhanced bioconversion performance through the disruption of the *giant* (*gt*) gene, a conserved transcription factor involved in growth regulation [67]. The resulting *gt* mutants exhibited significantly larger body size, faster larval growth, and a 13.1% increase in food waste conversion efficiency, while maintaining normal development and fertility. Molecular analyses revealed that *gt* loss-of-function upregulated *insulin-like peptide (ilps)* expression, promoting growth from early larval stages, and produced larvae with a thinner cuticle likely adapted to rapid growth. The mutants maintained stable nutrient composition and displayed higher yields across multiple waste substrates, including food, distiller’s grain, and vegetable residues. Importantly, competitive fitness assays demonstrated reduced reproductive competitiveness compared to wild type, indicating low ecological risk. This study provides one of the clearest demonstrations to date that targeted genome editing can directly and quantitatively improve BSF bioconversion performance, linking a single genetic modification to increased waste conversion efficiency across multiple substrates. As illustrated in Figure 1, advances in BSF genomics provide a mechanistic link between genetic diversity, trait optimisation, and bioconversion efficiency, ultimately supporting circular economy strategies and sustainable waste valorisation.

Table 1 provides an overview of current genetics insights and genome engineering advances on BSF to outline the emerging opportunities for applied biotechnology in this species.

## 5. Limitations, Regulatory Frameworks and Industrial Considerations

While recent advances in CRISPR/Cas9 and *piggyBac*-based genome editing have demonstrated substantial potential for improving BSF traits, several practical, regulatory and industrial considerations must be addressed to ensure successful deployment at scale. First, pleiotropic effects and fitness trade-offs may arise, whereby genetic modifications intended to enhance larval growth, substrate conversion efficiency, or nutrient composition could inadvertently affect reproductive capacity, stress tolerance, or survival under production conditions. For example, bearing in mind one of the studies referred above, gt mutants display increased larval growth and waste conversion efficiency but exhibit reduced reproductive competitiveness, which, while limiting reproductive fitness, may be advantageous in reducing ecological risk. This example marks the importance of balancing performance gains with safe deployment strategies. Moreover, the stability of engineered traits over successive generations under industrial-scale rearing conditions remains largely untested, highlighting the need for continuous monitoring and standardised protocols to maintain consistent performance.

Second, the translation of laboratory-scale genome editing advances to industrial production poses significant technical and economic challenges. Scaling genome-edited BSF lines requires maintaining genetic stability, uniform gene expression, and consistent phenotypic performance across large populations and multiple generations under intensive rearing conditions. Variability in substrate composition, environmental parameters, and rearing density may influence the expression of engineered traits, potentially reducing the predictability of performance gains observed under controlled experimental settings. Consequently, standardisation of rearing conditions, substrates, and performance metrics, together with harmonised phenotyping protocols, is essential to ensure reproducibility and comparability across facilities.

From an economic perspective, the benefits of genome editing must be carefully weighed against the costs and time requirements associated with regulatory approval, biosafety assessment, and infrastructure adaptation. Conventional selective breeding remains a well-established and comparatively low-cost approach for improving traits such as growth rate and substrate conversion efficiency. Genome editing may offer faster and more precise trait optimisation; however, its industrial adoption will depend on whether performance gains outweigh the additional financial and regulatory burdens. Comprehensive cost–benefit analyses comparing genome-edited lines with selectively bred strains are therefore required to assess their practical value for commercial production.

Furthermore, the successful deployment of genome-edited BSF depends on their integration into existing waste-management infrastructures. Industrial bioconversion systems must accommodate heterogeneous waste streams, variable supply chains, and regulatory constraints governing waste handling and feed production. Genome-edited BSF lines may require tailored rearing protocols or controlled environments to maximise performance, potentially limiting compatibility with decentralised or low-tech waste-management systems. Aligning genetic innovations with existing industrial processes, logistics, and regulatory frameworks is therefore critical to ensure scalability and economic viability within circular bioeconomy and waste valorisation strategies. Regulatory frameworks, biosafety, and public acceptance also represent key constraints on the use of genetically modified BSF. Regulatory requirements vary between regions. In the EU, genetically modified organisms (GMOs) are regulated under Directive 2001/18/EC and Regulation (EC) No 1829/2003 [68,69], which mandate comprehensive environmental risk assessments, approval for both feed and food use, and post-market monitoring. In practice, the EU generally maintains a restrictive stance on the cultivation of GMOs, with most Member States prohibiting or opting out of GMO production. Nevertheless, there are notable exceptions, such as the cultivation of insect-resistant genetically modified maize, which is authorised at EU level and currently grown in a limited number of countries, including Spain [70]. Insects genetically modified using CRISPR/Cas9, are currently classified as GMOs under EU law, meaning that even precise edits are subject to the same regulatory framework, potentially limiting or delaying industrial deployment. As stated above, while several GMO-derived products are authorised for use in food and feed in the EU, their approval is subject to exceptionally strict and precautionary legislation. In contrast, the use of genetically modified insects for non-food industrial applications would likely encounter a less stringent and more feasible regulatory pathway.

In contrast, several non-EU countries, including Brazil, the United States, India, Australia, Mexico, Costa Rica, among others, apply more permissive and product-oriented regulatory approaches to GMO cultivation [71].

Public acceptance also plays a critical role. In the EU, societal attitudes towards GMOs remain generally cautious, influenced by environmental, health, and ethical concerns, whereas in many non-EU regions public acceptance tends to be higher, particularly when genetically modified organisms are associated with clear agronomic, environmental, or sustainability benefits. Consequently, regulatory heterogeneity and public perception must be carefully considered when designing deployment strategies for genome-edited BSF, alongside biosafety measures, transparent risk assessment, and stakeholder engagement.

## 6. Future Perspectives and Conclusions

As stated above, recent studies in genomics and gene editing of the BSF have demonstrated the potential for optimising its biological traits, enhancing industrial performance and addressing biosafety concerns. The completion of a high-quality BSF genome assembly, together with the successful development of CRISPR/Cas9 and *piggyBac* transposase systems, brings researchers closer to having a genetic toolkit that will facilitate efficient and accessible modifications to the BSF genome. This advancement holds promises for the refinement of traits related to growth rate, nutrient assimilation, pathogen resistance, and environmental tolerance. The establishment of transgenic lines with traceable markers could further enhance process traceability and biosafety. Moreover, genetic modification may be employed to improve nutrient composition or to enable the biosynthesis of bioactive compounds, thereby increasing the nutritional and economic value of BSF-derived products. Despite these encouraging developments, challenges persist in achieving precise trait edition, and significant gaps remain in the ethical and regulatory frameworks required for the responsible use of genetically modified insects. Future research should prioritise the systematic identification of BSF genes that influence key industrial traits, using comparative genomics and functional studies informed by model organisms such as *Drosophila melanogaster* [72]. Homologous genes in *Drosophila* with well-characterised functions could guide the selection of candidate genes in BSF for targeted modification. Additionally, developing standardised protocols for efficient germline transformation will be critical to ensure reproducible and scalable genetic modifications.

Furthermore, research should investigate the integration of multi-trait engineering approaches, such as combining enhanced nutrient conversion with improved pathogen resistance or environmental stress tolerance, while maintaining overall fitness. Advances in synthetic biology, including modular genetic circuits and gene drives with controllable activation, may provide powerful tools for these purposes. Parallel efforts are needed to develop robust biosecurity and biocontainment strategies, ethical guidelines, and risk assessment frameworks that address both ecological and public health considerations.

Ultimately, integrating genetic improvement with sustainable by-product upcycling will consolidate the role of BSF as a model organism for the circular bioeconomy.

## Figures and Tables

**Table 1:** BSF key genetic features, genome manipulation and its relevance for industry.

Feature	Key Findings	Industrial Application	References
**Genetic origin**	South America: 52 haplotypes identified globally.	Identification of lineages with desirable traits for selective breeding and maintaining genetic diversity in industrial stocks.	[32,33]
**Genome** **composition**	Genome size: 1102 Mb; Number of genes: 16,770; mitochondrial genome length: 15,698 bp.	Provides reference for genomic studies and development of molecular tools to monitor or manipulate BSF populations.	[37,40]
**Affinity for** **organic matter**	326 genes expressed in larvae across different substrates; major genes involved in ribosome function and digestion. Gut microbiota varies between genetically distinct lines, indicating host genetics influences bioconversion efficiency.	Highlights genetic basis for substrate utilization; guides strain selection and optimisation for industrial bioconversion.	[37,46]
**Immune system/environmental adaptation**	31 PGRPs, 16 GNBPs, 57 AMP genes including cecropins and defensins, sarcotoxin and stomoxyn genes; phenoloxidase system; antimicrobial activity against Gram-positive/negative bacteria, fungi, viruses.	Ensures larval survival and biosafety during industrial-scale by-products conversion.	[18,19,37,50,51,52,53,54,55,56]
**Genome editing**	CRISPR/Cas9 and *piggyBac* systems enable targeted gene modifications and creation of transgenic BSF lines. Reported advances include *PTTH* mutation to extend the larval stage (larger larvae), *CarRA/CarB* insertion to produce carotenoids, and fluorescent lines for traceability. A recent binary CRISPR/Cas9 system using germline promoters generated stable Cas9-expressing lines with normal development, enabling highly efficient germline mutagenesis when crossed with sgRNA lines (e.g., *white*). Targeting vestigial produced wingless, sterile adults for safe, controllable mass rearing.	Enables optimisation of growth, nutrient content, and traceability in industrial bioconversion while providing biosafety controls.	[37,62,64,65,66]

## Data Availability

No new data were created or analyzed in this study. Data sharing is not applicable to this article.

## Abbreviations

The following abbreviations are used in this manuscript:

BSF	Black soldier fly
BSFL	Black soldier fly larvae
CRISPR/Cas9	Clustered Regularly Interspaced Palindromic Repeats/CRISPR-associated protein 9
EU	European Union
GMOs	Genetically modified organisms
